# Bacterial indicators of environmental stress in the gut microbiome of free-ranging European roe deer inhabiting agricultural landscapes

**DOI:** 10.1038/s41598-025-14933-w

**Published:** 2025-08-07

**Authors:** Rafał Łopucki, Ewa Sajnaga, Kinga Ożga, Dagmara Stępień-Pyśniak, Arkadiusz Jastrzębski, Marcin Świątek, Marta Kloch, Ilona Sadok, Paweł Nasiadka, Petter Kjellander, Daniel Klich

**Affiliations:** 1 Department of Biomedicine and Environmental Research, The John Paul II Catholic University of Lublin, Konstantynów 1J, Lublin, 20-708 Poland; 2 Department of Veterinary Prevention and Avian Diseases, University of Life Sciences in Lublin, Głęboka 30, Lublin, 20-612 Poland; 3 Department of Animal Breeding and Nutrition, Warsaw University of Life Sciences (SGGW), Ciszewskiego 8, Warsaw, 02-786 Poland; 4 Department of Animal Genetics and Conservation, Warsaw University of Life Sciences (SGGW), Ciszewskiego 8, Warsaw, 02-786 Poland; 5 Department of Biomedical and Analytical Chemistry, The John Paul II Catholic University of Lublin, Konstantynów 1J, Lublin, 20-708 Poland; 6 Department of Ecology, Grimsö Wildlife Research Station, Swedish University of Agricultural Sciences, Riddarhyttan, 73993 Sweden

**Keywords:** Bacteria, Cortisol, *Capreolus Capreolus*, Gut microbiota, Ruminants, Stress hormones, Ungulates, Ecology, Microbiology, Physiology

## Abstract

Environmental stressors can influence the gut microbiota of wild ruminants, yet their effects in free-ranging populations remain poorly understood. This study examined associations between physiological stress and gut microbiota composition in free-ranging European roe deer (*Capreolus capreolus*) from agricultural landscapes in central Poland. Fecal samples from 54 legally hunted individuals were analyzed for cortisol metabolite concentrations and bacterial community composition using 16S rRNA gene sequencing. Cortisol metabolite levels ranged from 17.9 to 371.4 ng ml⁻¹, allowing classification into low- and high-stress groups. Alpha diversity metrics did not differ between groups, but beta diversity analyses revealed significant differences in microbial community structure linked to stress. Stress remained a significant predictor of microbiota composition even after adjusting for confounding variables such as area and season, with its effect varying by context. Ratios of *Christensenellaceae* to *Rikenellaceae*,* Bacteroidaceae* and *Prevotellaceae* were significantly elevated in the high-stress group, indicating potential as microbial biomarkers of physiological stress. Additionally, *Barnesiellaceae* and *Succinivibrionaceae* (families involved in immune modulation and fermentation) were depleted under higher stress conditions. These findings highlight the role of gut microbiota in responses to environmental stressors and suggest that microbial signatures could serve as biomarkers for assessing the impact of agriculture on wildlife health and ecosystem stability.

**Supplementary Information:**

The online version contains supplementary material available at 10.1038/s41598-025-14933-w.

## Introduction

The mammalian gut hosts thousands of microbial species collectively referred to as the gut microbiota^1,2^. These microorganisms can play various roles in the host’s metabolism, influence nutrient absorption, regulate immune responses, and provide protection against pathogens^3^. The composition of the gut microbiota dynamically depends (directly and/or indirectly) on various internal factors and external environmental factors. The most significant internal factors include the host’s genotype and its current physiological and health status^4,5^. Among external factors, the most critical natural determinant directly affecting the gut microbiota is the type and availability of food^3,6,7^. However, other environmental factors should not be overlooked, as they may influence the microbiota indirectly through physiological mechanisms, such as responses to social stress, stress induced by predators or human activity or seasonality in food abundance and quality^8–10^.

Given that the relationship between vertebrates and their gut microbiota has been shaped over a long period of coevolution, it can be expected that the observed dynamics of microbiota in response to various environmental stimuli may have adaptive significance, allowing the host to adjust more effectively to temporary or permanent environmental changes^11–13^. Elucidating the patterns of this adaptation through the identification of specific microbial signatures associated with stressor factors could be crucial to gain deeper insights into the principles governing natural systems, develop new ecological indicators, and design tools for effective resource management. Unfortunately, despite an increasing number of studies in this field and continuous improvements in research methodologies, our knowledge of the complex relationship between complex gut microbial communities and their hosts remains limited^1,14^.

Notably, substantial knowledge gaps persist in the case of ruminants, for which the symbiotic microbiota is an essential key to accessing energy and nutrients from plant matter^15,16^. While microbiota dynamics in this group of animals have been primarily studied in domesticated livestock such as dairy cattle, beef cattle, and sheep^14^, there is a notable lack of data on wild, free-living ruminants, including cervid species. Studies in these animals remain limited to a few species and often relies on small sample sizes^3,7,13,14^. Yet, many species of wild ruminants play a crucial role in ecosystem functioning, wildlife management, and even commercial forestry^17^. Others are the focus of active conservation efforts, including translocations, reintroductions, and the development of optimal breeding strategies^3,18,19^. These interventions, while necessary, expose animals to a range of anthropogenic stressors that may disrupt physiological processes, including those mediated by the gut microbiota. Therefore, understanding the relationships between changes in gut microbiota composition and the stress factors driving it can be critical for making informed management decisions^1,17,18,20,21^. In this context, understanding how environmental stressors affect the gut microbial composition of cervids becomes particularly important. Such knowledge can inform conservation planning, improve animal welfare during management interventions, and offer insight into the ecological consequences of stress-induced microbial shifts^1,17,18,20,21^.

A contemporary summary of research on the ecological interactions of deer and their gut microbiota is presented in some studies^3,7,13,14,17^. These studies confirmed that the composition of deer gut microbiota is influenced by factors such as captivity, season, geographical location, weaning, and the presence of pathogens. However, as evidenced by these works, the potential role of stress as a contributing factor has not been considered. Stress can integrate various challenges faced by animals during specific periods and provides an overarching measure of their physiological response. Indicators of medium- or long-term stress are particularly valuable in such contexts, as they minimize or mitigate the influence of single or short-lived episodes^22^. For wild deer, studies that simultaneously measure stress hormone levels and assess qualitative and quantitative changes in gut microbiota would be especially insightful, enabling researchers to correlate the state of the microbiome with physiological levels of hormonal stress markers and identify the levels of stress that may lead to gut dysbiosis and subsequent physiological dysfunctions. Earlier, a similar approach was taken for the southern white rhinoceros^23^.

In this study, we aim to apply this research framework to the European roe deer (*Capreolus capreolus* Linnaeus, 1758), an ungulate species widely distributed across Europe in landscapes characterized by a mosaic of woodlands and farmland^24,25^. Carbillet et al.^26^ demonstrated that roe deer can successfully serve as a model species for studying the relationships between stress, immunity, and animal behavior. Additionally, roe deer is considered particularly sensitive to various types of negative stimuli^27^. For example, it has been shown that this ungulate exhibits elevated cortisol levels in areas dominated by human-related stressors compared to areas where natural stressors prevail^28^. Other studies also confirm that roe deer respond to environmental stressors with changes in spatial distribution and/or increased cortisol levels^29–34^. Furthermore, elevated glucocorticoid levels have been negatively correlated with body mass in free-ranging populations of this species^35^. Such observations suggest that the European roe deer could serve as a sensitive indicator of the impact of various anthropogenic factors on wildlife. However, the precise links between stress responses in roe deer and alterations in their microbiome affecting host’s health and behavior remain undocumented to date.

The aim of this study was to (1) determine the physiological cortisol levels observed in free-ranging European roe deer populations inhabiting the agricultural landscape of central Poland, and (2) assess whether elevated cortisol levels are associated with significant changes in α and/or β diversity of bacterial communities and the abundance of specific taxa. Additionally, the study sought to identify microbiological markers, that is, groups of bacteria that are either promoted or suppressed in the gut microbiota depending on cortisol levels. We hypothesized that the relationship between the roe deer microbiota and environmental stress levels would be pronounced, and that the observed associations would provide a better understanding of the physiological consequences experienced by wild animals in modern landscapes dominated by human activity.

## Methods

### Collection of fecal samples in the field

**Fig. 1:**
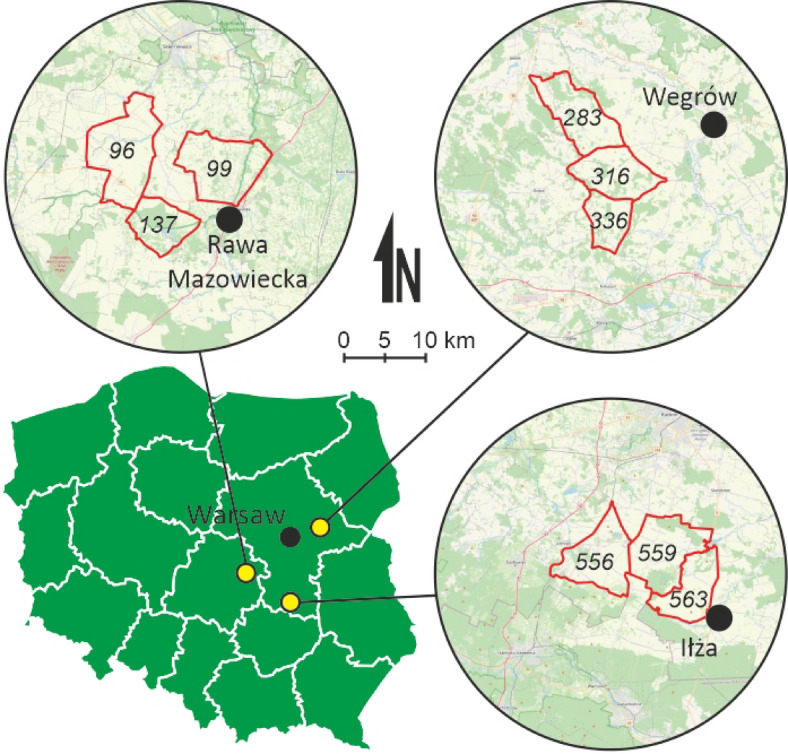
Location of study areas in Poland. The boundaries of the hunting districts (with identification number) from which the samples come are marked in red. The figure was generated in CorelDRAW Standard 2020 (https://www.corel.com), and the map background comes from National Geoportal service (https://mapy.geoportal.gov.pl/imap/Imgp_2.html).

The feces samples from roe deer were collected in central Poland (Europe), in the Mazowieckie and Łódzkie province, in the area of nine hunting districts, the smallest administrative game management units (Fig. 1) located in three study areas: Rawa Mazowiecka, Węgrów and Iłża. In those areas the hunting management is carried out and planned number of game animals (including roe deer) is harvested annually. In each hunting district, fecal samples were collected from an average of 6 (range 2–13) roe deer (Table 2). In total, 54 faecal samples from legally hunted roe deer were obtained for testing, i.e. 12 in Rawa Mazowiecka, 22 in Węgrów and 19 in Iłża.

Roe deer populations occurring in similar habitat conditions (agricultural landscape of central Poland) and subjected to similar hunting pressure were selected for the study to eliminate the influence of other factors on the microbiome as much as possible. A similar share of wind farms was included in each study region, in accordance with the findings of^34^.

**Table 1:** The number of fecal samples from roe deer collected in a specific hunting district.

Study areas	Hunting district identification number	Number of samples	Percent of samples
Rawa Mazowiecka	96	7	13
	99	3	5.6
	137	3	5.6
Węgrów	283	13	24
	316	7	13
	336	2	3.7
Iłża	556	5	9.3
	559	6	11
	563	8	14.8
Total		54	100

Fecal samples were collected per rectum during the autopsy of hunted individuals. The amount of feces collected (> 10 g) was sufficient to allow measurements of cortisol metabolites and bacterial microbiota tests, along with possible repetitions. The collected samples were kept on ice in a portable refrigerator and, as soon as possible (within 2–4 h), fecal samples were frozen and stored at −20 °C until further analysis. The samples were collected from each individual using new or disinfected instruments. This method of sampling ensured the acquisition of qualitatively uniform fresh material and an accurate description of the sex and carcass weight of the animal from which the fecal sample was taken (41 samples from females and 13 samples from males) (Table S1). The samples were collected in 2022–2024. Due to the adopted methodology, the samples were collected during the roe deer hunting season, which in Poland lasts from 11 May to 15 January.

### Assessment of cortisol metabolites in feces

Fecal cortisol metabolite levels in roe deer were analyzed using the commercial 11-oxoetiocholanolone ELISA kit (Item No. 501420; Cayman Chemical, Michigan, USA), which was validated by the manufacturer for use with ungulate feces. 11-oxoetiocholanolone is a cortisol metabolite found in animal feces, including ungulates^36^. The utility of 11-oxoetiocholanolone for assessing stress levels in cervids, including roe deer, has been previously validated^28,37,38^ and its effectiveness in evaluating the impact of both anthropogenic and natural stressors in wild populations have been demonstrated. The detection limit of the assay was 0.11 ng/ml, with an assay range of 0.061–1,000 ng ml⁻¹ and a sensitivity of 0.2 ng ml⁻¹. The test was carried out following the manufacturer’s instructions available on the manufacturer’s website at https://www.caymanchem.com/product/501420/11-oxoetiocholanolone-elisa-kit. Briefly, the procedure involved lyophilizing samples, weighing 0.05 g of dry mass, extracting cortisol metabolites with acetic acid and ethyl acetate, and reconstituting the supernatant in 500 µl of assay buffer. The samples were applied to a 96-well plate coated with anti-rabbit IgG, with specific reagents added, followed by overnight incubation. The wells were washed, pNPP substrate was added and the samples were incubated for 100 min. The yellow product’s absorbance at 405 nm, measured spectrophotometrically, was inversely proportional to free 11-oxoetiocholanolone concentration. Each sample was analyzed in duplicate, and results near the assay limits were reanalyzed after adjustment. The final values used for calculations were expressed in ng ml⁻¹. For each plate, an individual standard curve consisting of 8 points in duplicate was prepared.

### DNA extraction and 16S RNA amplicon sequencing

After thawing, each fecal sample was homogenized thoroughly using a sterile spatula and 0.2 g each was transferred to a bead tube. Metagenomic DNA was extracted using a PureLinkMicrobiome DNA Purification Kit (Invitrogen, Carsband, USA) according to the manufacturer’s recommendations (stool sample protocol). Assessing of isolated DNA concentration was carried out with Qubit2 fluorometer (Invitrogen, Carsband, USA) using a Qubit dsDNA HS Assay Kit (Thermo Fisher Scientific, Waltham, USA). The amplification of the V3-V4 region of the 16S rRNA gene was done using 338F: 5’- ACTCCTACGGGAGGCAGCA-3’ and 806R: 5’-GGACTACHVGGGTWTCTAAT-3’ primers, tailed with sample-specific Illumina index sequences to allow for deep sequencing by BMKGene (Biomarker Technologies, Beijing, China). The PCR was performed with KOD FX Neo DNA polymerase (TOYOBO Biotech, Shanghai, China) and the amplified DNA was then purified with the Omega DNA Purification Kit (Omega Inc., Norcross, GA, USA) and quantified using Qsep-400 (BiOptic, Inc., New Taipei City, Taiwan, ROC). Subsequently, 16S rRNA gen amplicon sequencing and bioinformatics analysis were performed by the same company. Sequencing was performed on a NovaSeq 6000 platform (Illumina, San Diego, USA) with paired-end mode (2 × 250). Sequencing data were deposited in NCBI under accession number PRJNA1187881 (https://www.ncbi.nlm.nih.gov/bioproject/PRJNA1187881/).

### Exploratory sequencing data analysis

Bioinformatics analysis was performed using BMKCloud (http://www.biocloud.net/). Raw data were filtered by Trimmomatic (ver. 0.33)^39^, while removal of primer sequences was processed by Cutadapt (ver. 1.9.1)^40^. Paired-end reads were assembled by USEARCH (ver. 10) and followed chimeric sequences detection and removal using UCHIME (ver. 8.1)^41,42^. Amplicon sequence variant (ASV) inference and denoising were performed using the DADA2 pipeline (ver. 1.22.0) in R^43^. The core parameters were: truncLen = 0, trimLeft = 0, maxEE = 2, truncQ = 2, poolMethod = independent, chimeraMethod = consensus, minParentFold = 4.0, and minoverlap = 10. These settings were chosen specifically to address the known limitations of NovaSeq-generated data, particularly the use of binned quality scores. Expected error filtering was prioritized over quality truncation to compensate for reduced resolution of per-base quality values. The taxonomy assignment was performed using the SILVA database (ver. 138.1)^44^ based on the Naive Bayes classifier in QIIME 2^45,46^.

Rarefaction curve analysis was performed using phyloseq in R^47^. To visualize the overlap of ASVs between groups of roe deer with differing fecal cortisol metabolite concentrations, the VennDiagram package in R (ver. 4.5.0) was used^48^. The *α*-diversity metrics, including Shannon, Simpson indices, and differences in these metrics between experimental groups (with *t*-test) were evaluated and visualized in Past (ver. 4.03)^49^.

### Assessment of microbiota indicators

To identify microbial indicators reflecting stress-related alterations in gut microbiota composition, we constructed abundance ratios between the phyla Bacillota and Bacteroidota (former Firmicutes/Bacteroidota ratio, F/B), although, recent large-scale meta-analyses have cast doubt on its reliability^50^. However, we did not limit our analysis to the phylum level; instead, we further investigated potential differences by calculating the indices at the family level. We first filtered the dataset to retain only taxa meeting strict prevalence (90%) and abundance (belonged to the top 10% most abundant families) thresholds to select only the most abundant and probably biologically relevant taxa. Next, we manually reviewed taxonomic assignments and excluded unclassified groups and ambiguous clades to retain only well-defined bacterial families with unambiguous taxonomic labels. We then computed pairwise abundance ratios between all Bacillota and Bacteroidota families using log2-transformed values with a pseudocount (+ 1) to handle zeros. Ratios of the Bacillota family to Bacteroidota family were tested for differences between high-stress and low-stress groups using the Wilcoxon rank-sum test. All analyses were conducted in R (ver. 4.5.0) using the following packages: readxl^51^, dplyr^52^, tidyr^53^, stats, and graphics for generating boxplots^54^.

### The β diversity analysis of gut microbiota

To assess the effects of multiple host-related factors on gut-microbial community structure, we applied PERMANOVA with the function adonis2 (vegan) on Bray-Curtis dissimilarities. We ran each model twice: (1) with the default setting by = NULL, which yields total sums of squares and thus overall model statistics, and (2) with by = “margin”, which returns marginal sums of squares and therefore the unique (partial) contribution of every individual predictor.

We first evaluated the effect of each predictor on its own (Stress, Sex, Area, Season and Weight) to identify variables that significantly explained β-diversity. Then, we built a candidate set of additive and two-way interaction models comprising all possible combinations of two and three variables (e.g. Stress + Area, Stress × Area, Stress + Area + Weight). Because only one high-stress individual was sampled in summer, the Stress × Season interaction and any higher-order terms containing it were excluded to avoid confounding. Each model was tested with 9 999 permutations. For every model we report both the overall fit (Total R², Total F, and *p*) and the marginal statistics for each term, allowing simultaneous comparison of global explanatory power and the unique effect of each factor. Analyses were carried out in R 4.5.0 with the packages phyloseq^47^, vegan^55^ and base/utils^54^. Additionally, assessment of multivariate dispersion using PERMDISP2 were conducted, using the packages vegan^55^ and stats^54^ (R, ver. 4.5.0). These analyses revealed no significant differences in dispersion among groups defined by Area (*p* = 0.668), Stress (*p* = 0.689), Season (*p* = 0.256), or Sex (*p* = 0.306). These results indicate that the assumption of homogeneity of group dispersions was met for all categorical variables tested.

Non-metric multidimensional scaling (NMDS) was used to visualize differences in microbial community composition between roe deer with higher and lower cortisol levels. The analysis was based on Bray–Curtis dissimilarity using the vegan^55^ package in R (ver. 4.5.0). Ordination was performed with metaMDS() using two dimensions (k = 2) and 100 random starts (trymax = 100). The final solution had a stress value of 0.179, indicating an acceptable representation of the data in reduced dimensionality. Ellipses representing the 90% confidence intervals of each stress group were added (ggplot2 package, R ver. 4.5.0)^56^.

### Statistical testing of differential abundance

Differential abundance analysis of the gut microbiota was performed using the ANCOM-BC2 (Analysis of Composition of Microbiomes with Bias Correction 2) framework, which accounts for compositionality and sampling bias in high-throughput sequencing data. The primary objective was to identify bacterial families whose relative abundances differed significantly between roe deer exhibiting higher versus lower stress levels. Additional covariates included three study area (Area: IŁŻA, RAWA, WĘGRÓW), Sex (F - Female and M - Male), Season (Summer and Winter), and carcass weight (Weight) (Table S1). For each model, ANCOM-BC2 was applied using the ancombc2() function in R (ver. 4.5.0)^57^. Statistical significance was assessed using FDR-adjusted p-values (q-values), with a significance threshold of q < 0.05. Taxa were filtered based on minimum prevalence (≥ 5% of samples) and library size (≥ 1,000 reads per sample). In models including multilevel categorical covariates (Area), pairwise comparisons were enabled to assess group-specific effects. Structural zero detection was applied where applicable. In addition to statistical significance (q-value < 0.05), signal sensitivity (ss) was assessed to evaluate the robustness of differential abundance findings. This metric reflects the extent to which the observed signal is stable across resampling perturbations or model noise, and is used to filter out results that are statistically significant but biologically unstable or unreliable. Only taxa that passed both the adjusted significance threshold and demonstrated adequate signal sensitivity were considered differentially abundant and marked as “TRUE”. For each model, significant taxa were extracted, and the number of differentially abundant families per model was summarized.

### Ethical statement

None of the animals were intentionally killed for the purposes of this study. Samples were occasionally obtained from legally hunted individuals through cooperation with hunting clubs under a research grant.

## Results

### Fecal cortisol metabolites concentration

The 11-oxoetiocholanolone concentrations measured in the 54 roe-deer faecal samples ranged from 17.9 to 371.4 ng ml⁻¹ (Table S1). Because the Bimodality Coefficient indicated a tendency toward a bimodal distribution (BC = 0.58), we divided the material into two subsets. To keep the groups approximately equal in size for downstream statistics, we used the sample median (107 ng ml⁻¹) as the cut-off. Animals with metabolite concentrations ≤ 107 ng ml⁻¹ were assigned to the Low-stress group (*N* = 28), whereas those with concentrations > 107 ng ml⁻¹ formed the High-stress group (*N* = 26). The Low-stress group averaged 57.9 ± 28.8 ng ml⁻¹, while the High-stress group averaged 205.8 ± 75.1 ng ml⁻¹. All subsequent microbiota analyses were carried out using this Low/High categorization.

To assess the robustness of our findings, we additionally conducted a PERMANOVA in which fecal cortisol metabolite concentration was treated as a continuous predictor (log₁₀-transformed). This analysis used the same Bray–Curtis dissimilarity matrix and was performed using the adonis2() function from the vegan R package (ver. 4.5.0) with marginal sums of squares (by = “margin”) and 9,999 permutations. Results are summarized in Table S4.

### 16S rRNA gene sequencing results

**Fig. 2:**
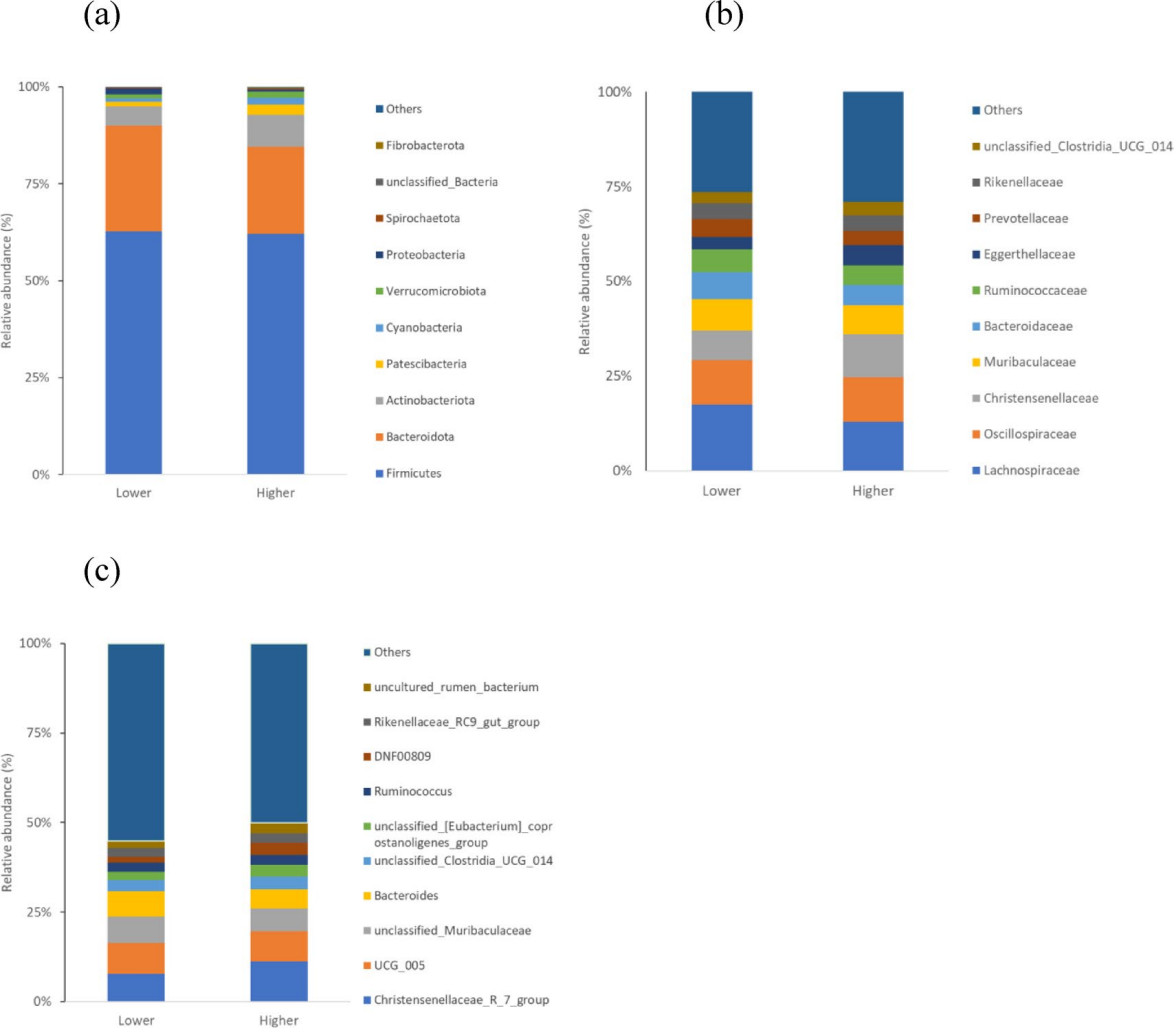
Relative abundance of 10 top bacterial taxa at the phylum (**a**), family (**b**) and genus (**c**) level in the gut microbiota of roe deer differing in fecal cortisol level. Abbreviations: Lower – the group with low concentration of cortisol metabolites, Higher – the group with elevated concentration of cortisol metabolite, *N* = 28 and *N* = 26, respectively.

For both groups, the number feature sequences passing quality filtering in the samples ranged from 92 356 to 142 477 (median 119 937) (Table S2). All rarefaction curves converged to a horizontal asymptote, demonstrating that the sequencing data were sufficient to describe the bacterial diversity in the samples (Fig. S1). The selected ASV ranged from 559 to 2811 for individual samples (median 1207) (Table S2). The number of specific ASVs was 19 179 and 21 175 for the group with low and high stress, respectively, exceeding the number of shared ASVs (4920) (Fig. S2). The summary of the assignment of ASV to the bacterial taxonomy is presented in Table S3. The most abundant taxa in each group are illustrated in Fig. 2.

### Microbiota indicators

**Fig. 3:**
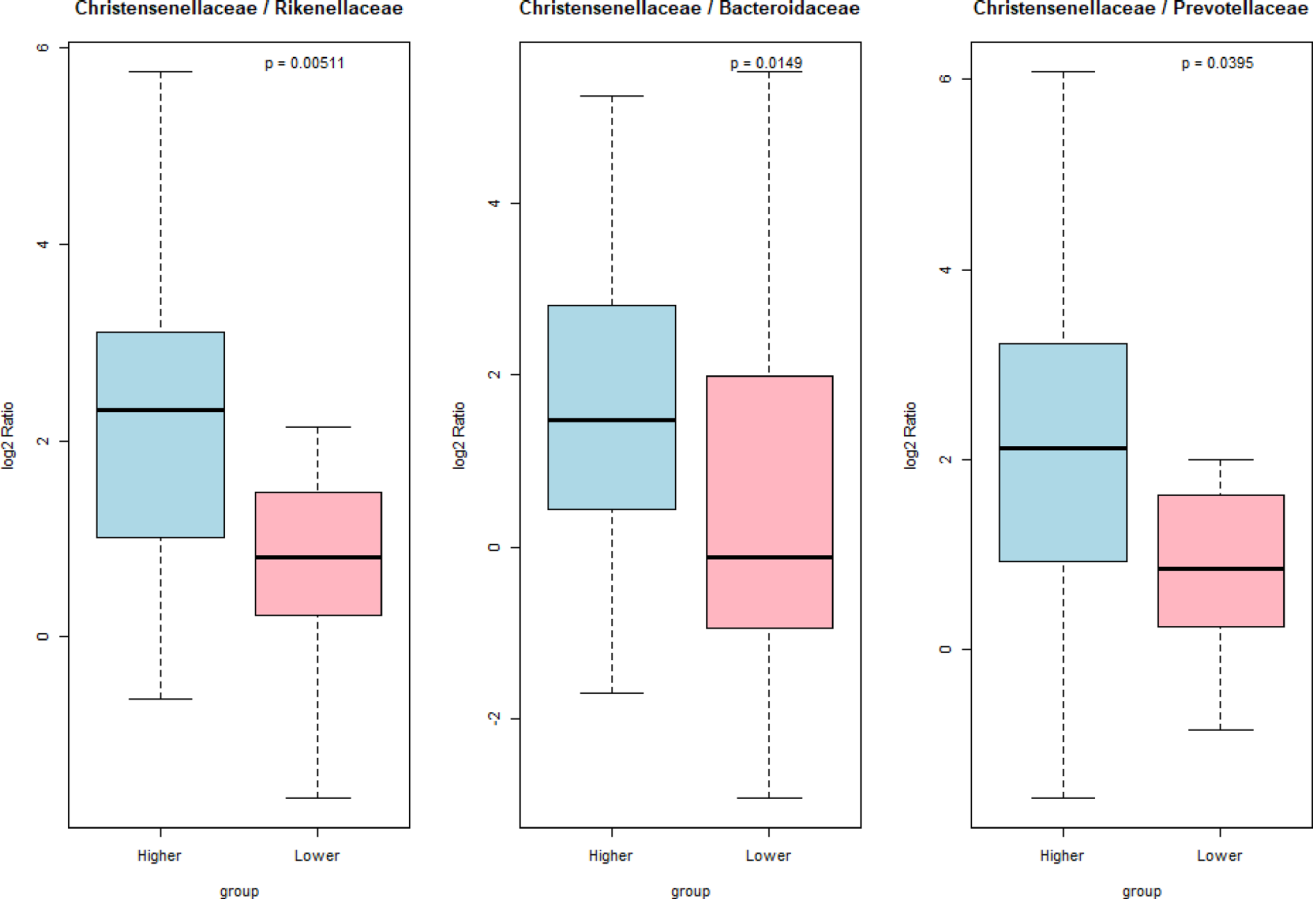
Boxplots showing log2-transformed abundance ratios of *Christensenellaceae* (Bacillota) to selected Bacteroidota families in the gut microbiota of roe deer categorized into Higher and Lower stress groups. The ratios represent *Christensenellaceae* relative to *Rikenellaceae* (left), *Bacteroidaceae* (center), and *Prevotellaceae* (right). Significance levels are indicated directly on the plots. Data processing and visualization were performed in R (ver. 4.5.0) using the readxl, dplyr, and base graphics packages. Statistical comparisons were carried out using the Wilcoxon rank-sum test (stats package).

The Bacillota/Bacteroidota ratio (former Firmicutes/Bacteroidota ratio) did not differ significantly between the Low and High stress groups (U = 280; z = 1.44; *p* = 0.15). Given that this ratio aggregates phylum-level abundances and may overlook relevant compositional shifts at finer taxonomic resolution, we additionally examined taxon ratios at the family level. Three microbial indicators involving the family *Christensenellaceae* (phylum Bacillota) in relation to selected Bacteroidota families showed significantly higher values in the Higher stress group: *Christensenellaceae vs. Rikenellaceae* (*p* = 0.005), *Christensenellaceae vs. Bacteroidaceae* (*p* = 0.015), and *Christensenellaceae vs. Prevotellaceae* (*p* = 0.039) (Fig. 3).

### The α and β diversity of gut microbiota

The *α*-diversity referring to the observed richness and evenness of the microbiota samples was assessed using Simpson (D) and Shannon (H) indices (Low stress group: D = 0.98, H = 5.21; High stress group: D = 0.98, H = 5.40), however, no statistically significant differences were observed between the studied groups (Fig. S3).

Permutational multivariate analysis of variance (PERMANOVA) based on Bray–Curtis dissimilarity identified several statistically significant effects of host-related variables on gut microbial community composition (Table 2). Among individual predictors, Area explained the largest proportion of the variation in gut microbial composition (Total R² = 0.120, *p* < 0.001), followed by Stress (R² = 0.058, *p* = 0.005) and Season (R² = 0.043, *p* = 0.030), whereas Weight was not significant (R² = 0.013, *p* = 0.743) (Table 2).

**Table 2:** Results of PERMANOVA assessing the effects of four selected factors (Area, Stress, Season, and Weight) and their interactions on beta diversity (Bray–Curtis distance) of the gut microbiota in European roe deer. The table includes all models for which a statistically significant effect was observed (*p* < 0.05). For each model (full formula fitted in PERMANOVA), the following parameters were presented: total R² (variance explained by the entire model), total F (F-statistic for the whole model), model *p* (permutation *p*-value for the whole model), term (name of the individual factor or interaction being tested), Df (degrees of freedom for the term), SS partial (marginal sum of squares for the term), R² partial (unique proportion of variance explained by the term), F partial (F-statistic for the term after accounting for others) and term *p* (permutation *p*-value for the term). In interaction models, only the highest-order interaction term is presented.

Model	Total *R*²	Total F	Model *p*	Term	Df	SS (partial)	R2 (partial)	F (partial)	Term *p*
Stress	0.058	3.198	0.005						
Area	0.120	3.473	< 0.001						
Season	0.043	2.342	0.030						
Weight	0.013	0.660	0.743						
Stress + Area	0.164	3.279	< 0.001	Stress	1	0.199	0.045	2.664	0.014
				Area	2	0.475	0.106	3.186	0.001
Stress + Season	0.085	2.359	0.008	Stress	1	0.186	0.042	2.316	0.025
				Season	1	0.119	0.027	1.490	0.139
Stress + Weight	0.073	2.007	0.020	Stress	1	0.270	0.060	3.325	0.005
				Weight	1	0.067	0.015	0.827	0.565
Area + Season	0.158	3.131	< 0.001	Area	2	0.514	0.115	3.417	< 0.001
				Season	1	0.171	0.038	2.274	0.030
Area + Weight	0.131	2.517	0.001	Area	2	0.530	0.119	3.415	< 0.001
				Weight	1	0.051	0.011	0.653	0.744
Stress + Area + Season	0.190	2.877	< 0.001	Stress	1	0.143	0.032	1.939	0.057
				Area	2	0.471	0.106	3.193	< 0.001
				Season	1	0.115	0.026	1.561	0.121
Stress + Area + Weight	0.177	2.632	< 0.001	Stress	1	0.204	0.046	2.718	0.012
				Area	2	0.464	0.104	3.093	0.001
				Weight	1	0.056	0.012	0.743	0.644
Stress + Season + Weight	0.099	1.839	0.018	Stress	1	0.195	0.044	2.429	0.025
				Season	1	0.118	0.026	1.467	0.157
				Weight	1	0.066	0.015	0.817	0.574
Area + Season + Weight	0.170	2.501	< 0.001	Area	2	0.509	0.114	3.360	< 0.001
				Season	1	0.171	0.038	2.260	0.031
				Weight	1	0.051	0.011	0.671	0.736
Stress * Area	0.207	2.506	< 0.001	Stress: Area	2	0.190	0.043	1.289	0.192
Stress * Weight	0.094	1.739	0.023	Stress: Weight	1	0.096	0.022	1.188	0.266
Area * Season	0.195	2.326	< 0.001	Area: Season	2	0.165	0.037	1.099	0.326
Area * Weight	0.165	1.901	0.003	Area: Weight	2	0.152	0.034	0.980	0.445
Stress * Area * Weight	0.286	1.532	0.007	Stress: Area: Weight	2	0.103	0.023	0.681	0.819
Area * Season * Weight	0.281	1.682	0.002	Area: Season: Weight	1	0.055	0.012	0.735	0.665

Additive models combining multiple predictors demonstrated greater explanatory power. Stress appeared in most of the statistically significant models, suggesting that it accounted for a substantial proportion of the observed variation in *β*-diversity. For example, the additive model Stress + Area accounted for 16.4% of the variance (Total F = 3.28, *p* < 0.001). Within that model the unique (marginal) effect of Area and Stress remained significant: partial *p* = 0.001 and *p* = 0.014, respectively. Three-factor additive models produced only modest additional gains. For example, Stress + Area + Season increased Total R² to 0.190, yet the partial effect of Season became non-significant (*p* = 0.121). Interactions were generally uninformative: none of the two- or three-way interaction terms reached significance (all partial *p* ≥ 0.19), even though the corresponding full models sometimes exhibited high Total R² (e.g. Stress × Area × Weight, R² = 0.286).

Sex was excluded from the final PERMANOVA models based on both statistical and biological considerations. Univariate analysis showed that Sex was not a significant predictor of *β*-diversity (F = 1.09, R² = 0.021, *p* = 0.334), whereas Season had a significant effect (*p* = 0.030). Including Sex in multivariate models resulted in only a marginal increase in explained variance (ΔR² = 0.0182) and did not improve model performance. Moreover, due to wildlife harvesting practices (males are predominantly hunted in summer, whereas females and fawns in winter) Sex and Season are partially confounded. As a result, Season captures a broader range of biological and environmental variation, making it a more informative predictor. Excluding Sex not only simplifies the model without compromising explanatory power but also substantially reduces the number of models presented, improving clarity and interpretability.

**Fig. 4:**
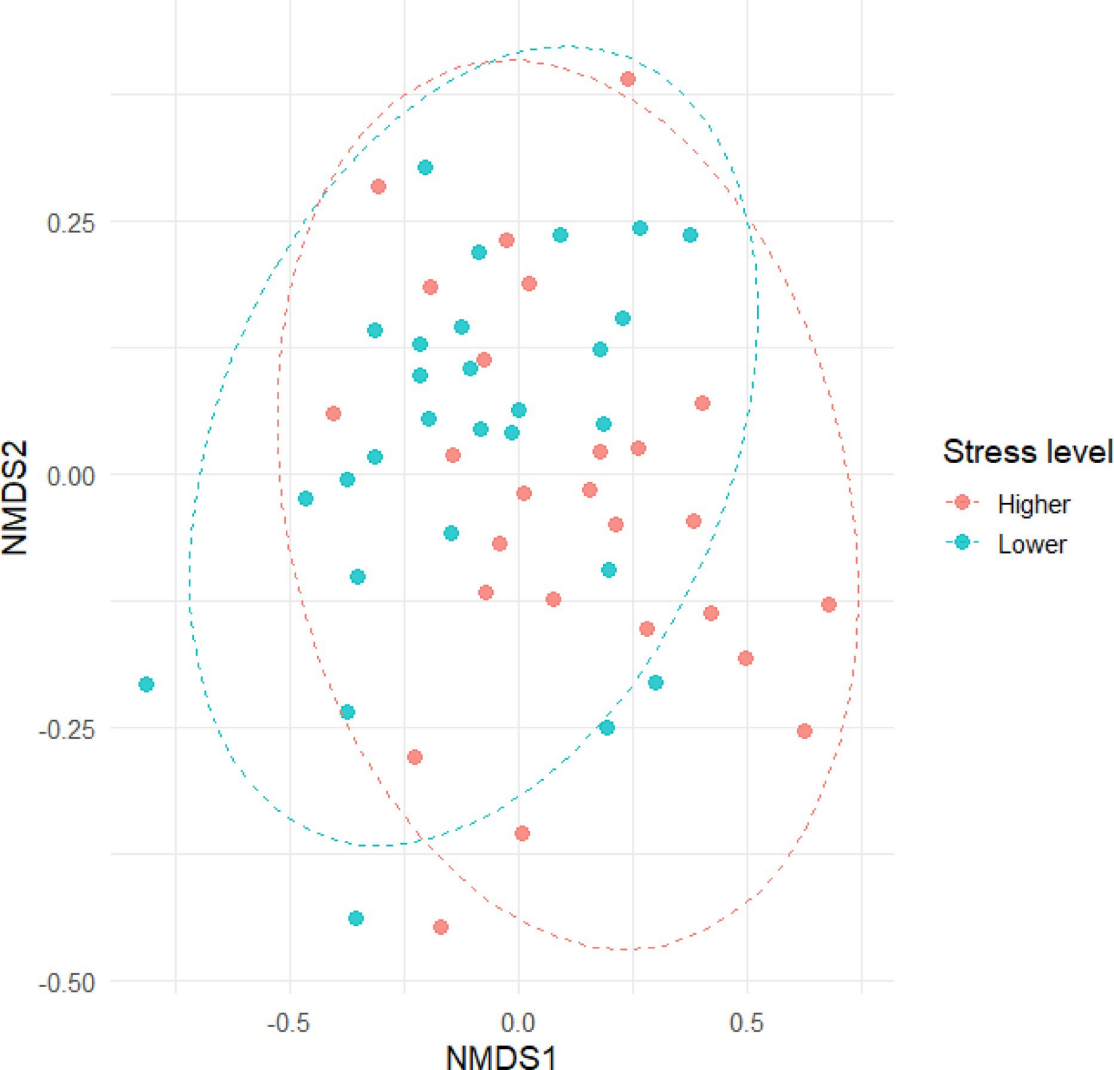
Beta diversity of gut microbiota at the family level in 54 European roe deer (Capreolus capreolus) classified into lower- and higher-stress groups based on fecal cortisol metabolite concentrations. The plot shows results of a non-metric multidimensional scaling (NMDS) analysis based on Bray–Curtis dissimilarities, illustrating differences in bacterial community structure between stress groups. Each point represents one individual, with ellipses indicating 90% confidence intervals for each stress level (red: higher stress; blue: lower stress). The configuration had a stress value of 0.178.

To visualize group-level differences, NMDS ordination based on Bray–Curtis distances was performed (Fig. 4). Samples grouped by stress level showed partial separation along the NMDS axes, with individuals from the High and Low stress groups occupying overlapping but distinct regions in ordination space. Ellipses representing 90% confidence intervals further illustrate the spread of samples within each stress category. The configuration had a stress value of 0.178, which is within the acceptable range for ecological data (< 0.2).

Comparative analyses using stress as a continuous variable (11-oxoetiocholanolone concentration - logConc) yielded patterns consistent with those from the binary approach (Table S4). These results indicate that how stress is parameterized (continuous vs. categorical) may influence statistical power, yet its biological relevance is evident across models. Although the binary grouping showed a stronger individual effect, continuous stress remained near-significant as a standalone predictor (*p* = 0.07) and consistently contributed to the most explanatory additive models: logConc + Area + Season (*p* < 0.001), logConc + Area (*p* < 0.001), and logConc + Area + Weight (*p* < 0.001). Notably, the categorical models also tended to exhibit higher total R² values, suggesting that dichotomizing stress may capture stronger or more nonlinear shifts in microbial composition. These findings together support the interpretation that stress is a robust driver of β-diversity regardless of representation. Notably, the continuous analysis also clarified the role of Season. While Season was not significant in the binary model (*p* = 0.139), its marginal effect became stronger when paired with logConc (*p* = 0.088), indicating a potentially independent contribution. Although not formally significant, this consistent pattern across models supports retaining Season as a biologically relevant covariate, interpreted with appropriate caution.

### Differential abundance of bacterial taxa

The ANCOM-BC2 analysis identified several bacterial families whose relative abundances differed significantly between groups defined by stress level, season, sex, and study area (FDR-adjusted q < 0.05; passed signal sensitivity criterion).

**Fig. 5:**
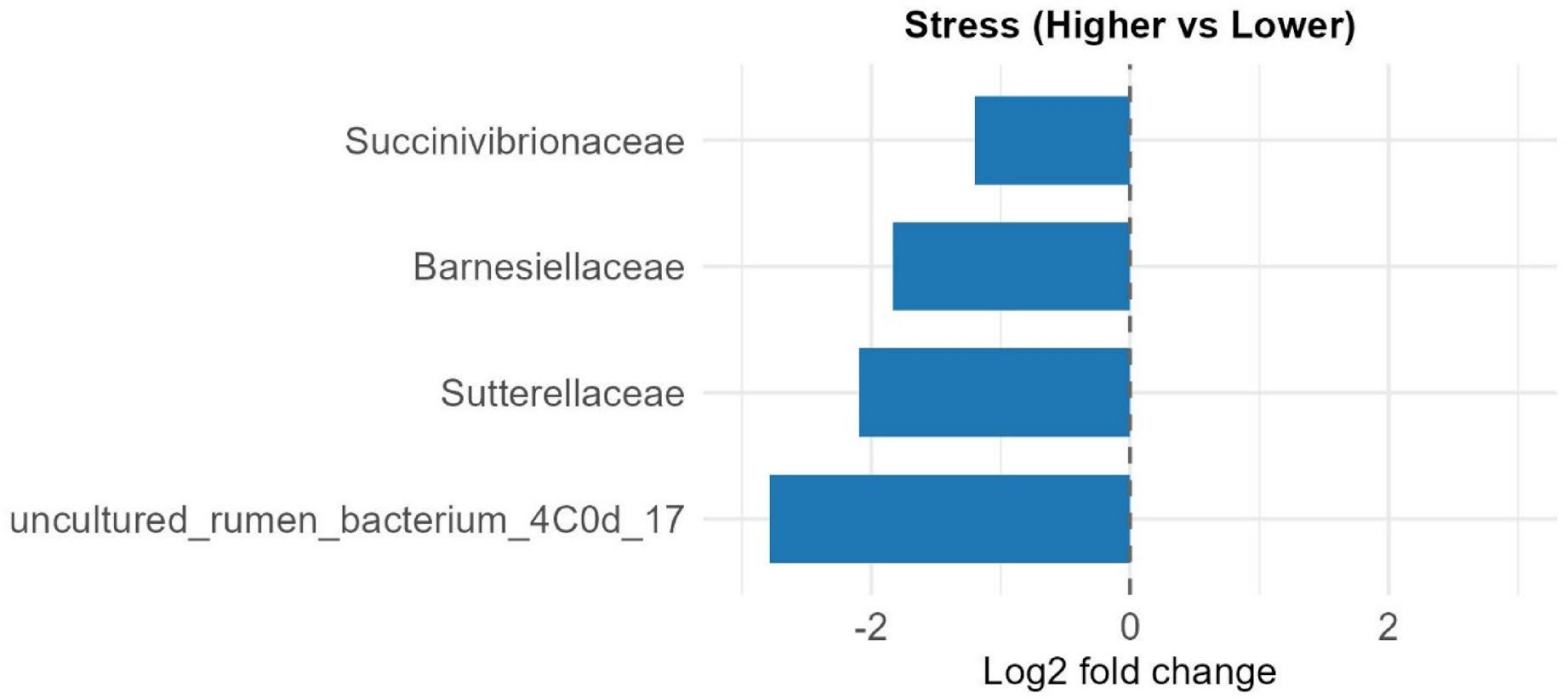
Differentially abundant bacterial families between roe deer with higher and lower stress levels. Bar plot shows taxa identified as significantly different in relative abundance (FDR-adjusted q < 0.05, passed signal sensitivity threshold) by ANCOM-BC2. All taxa displayed were significantly less abundant in individuals with higher cortisol levels. Bars represent log2 fold changes in relative abundance between stress groups (Higher vs. Lower).

**Fig. 6:**
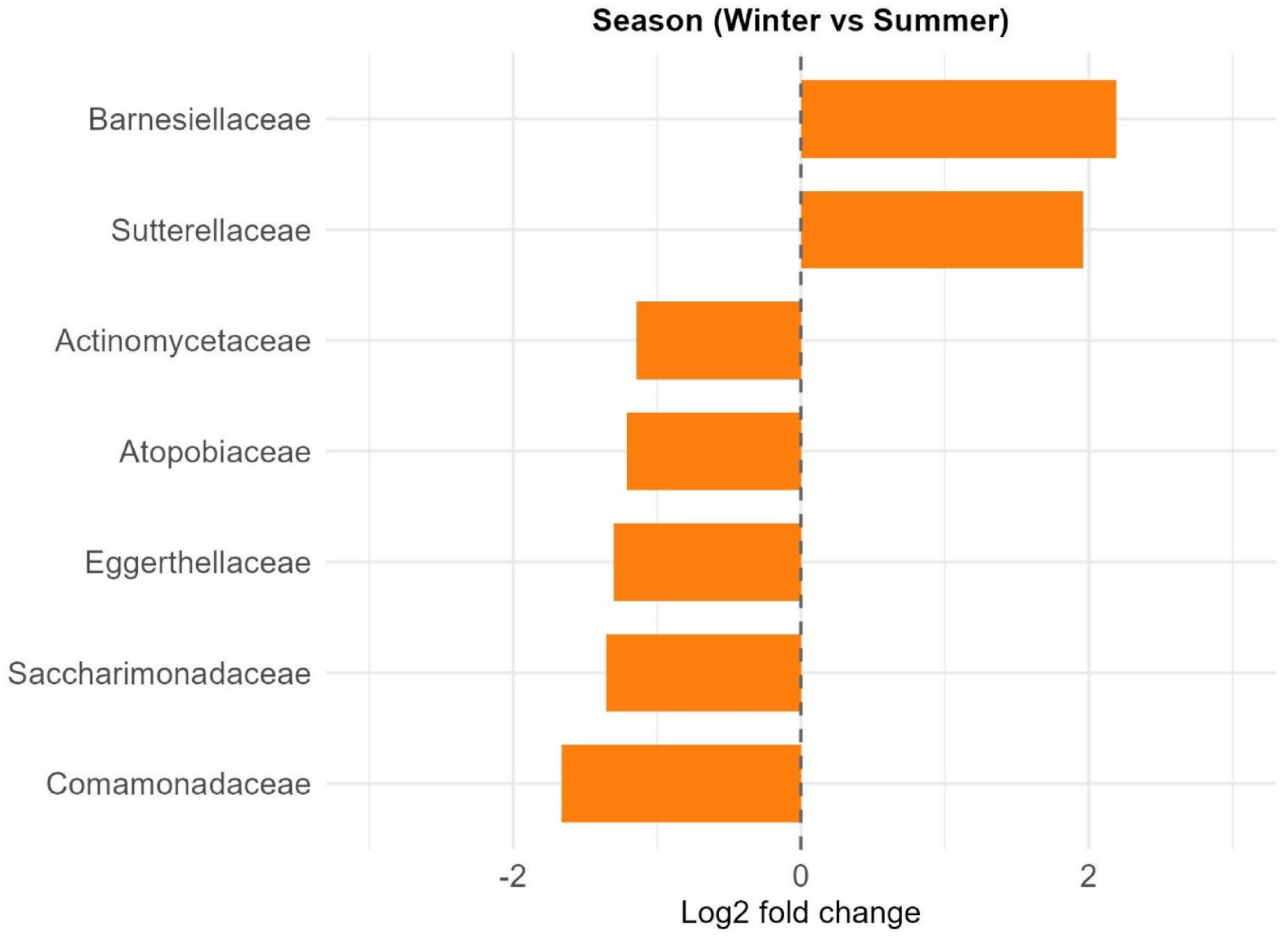
Seasonal variation in bacterial family-level composition of the roe deer gut microbiota. Bar plot shows families with significantly different relative abundances between winter and summer samples as identified by ANCOM-BC2 (FDR-adjusted q < 0.05; passed signal sensitivity threshold). *Barnesiellaceae* and *Sutterellaceae* were more abundant in winter, while the remaining taxa were more abundant in summer. Bars represent log2 fold changes (Winter vs. Summer).

For the Stress variable, four bacterial families exhibited significantly lower relative abundance in individuals with high stress levels compared to those with low stress. These included *Barnesiellaceae*, *Succinivibrionaceae*, *Sutterellaceae*, and an uncultured rumen bacterium (4C0d_17) (Fig. 5). Regarding Season, seven families showed significant seasonal shifts. Relative abundances of *Actinomycetaceae*, *Atopobiaceae*, *Comamonadaceae*, *Eggerthellaceae*, and *Saccharimonadaceae* were significantly reduced in winter compared to summer, whereas *Barnesiellaceae* and *Sutterellaceae* were more abundant in winter (Fig. 6). For Sex, only a single taxon, uncultured *Paenibacillaceae* bacterium, exhibited significantly lower abundance in males compared to females (lfc − 1.294, q = 0.029). In terms of geographic area, comparisons between Iłża vs. Węgrów (Fig. S4) and Rawa vs. Węgrów (Fig. S5) revealed increased abundances of 12 and 9 bacterial families in samples from Węgrów, respectively. No significant differences were observed between Iłża and Rawa.

## Discussion

As reported in numerous studies, stress is a significant factor influencing the composition of gut microbiota^58^, yet this phenomenon remains poorly understood in cervid species^3^. Wild cervids are exposed to a variety of environmental stressors, including severe climate, seasonality in food abundance and quality, natural predation and human hunting^27,59–61^, which imply that microbiota responses to these stressors might also be expected in these animals. On the other hand, evolutionary processes should have led to the development of adaptive mechanisms that mitigate the microbiota’s stress response and prevent harmful gut dysbiosis. Our research likely showed such an adaptive phenomenon - a reduction in the abundance of certain bacterial groups sensitive to elevated cortisol levels, with their functions being replaced by other microbial groups.

In our study, we focused on physiologically natural stress levels observed in free-ranging roe deer populations inhabiting a lowland agricultural landscape. Consequently, we did not examine extreme cases of elevated stress and/or gut dysbiosis, such as those reported in large mammals subjected to experimental stress scenarios, including prolonged transportation^9,62^, or those induced intentionally in laboratory animals^63,64^. However, the wide range of concentrations of 11-oxoetiocholanolone in the studied roe deer allowed them to be classified into two groups, differing in stress level. Importantly, we did not aim to determine the specific causes of the observed stress. Instead, our primary interest was to provide a general characterization of stress levels within the population and examine their association with microbiota composition. It is essential to note that both cortisol secretion and microbiota changes can exhibit considerable dynamism, with even substantial effects potentially compensated by the organism within a few days^65^ or rarely longer^9^. Conducting in-depth and detailed microbiota studies on wild populations of ruminants poses challenges due to the difficulty of controlling all relevant variables and assembling an adequately sized research sample. For this reason, the identification of broad and universal indicators may represent a more practical and effective approach.

An example of such an indicator is the Bacillota to Bacteroidota (B/B, former F/B), ratio calculated at the phylum level^3,7^. The reliability of this ratio has been called into question^50^, but Sun et al.^66^ suggest that in ruminants, as in humans, a higher B/B ratio may be associated with increased energy harvest from colonic fermentation and enhanced production of short-chain fatty acids (SCFAs). In accordance with this, under conditions of elevated stress, higher values of the B/B ratio can be expected, as stress is typically associated with the need for organismal mobilization and more energy-intensive metabolism. However in our study, groups of microbiota of roe deer differing in cortisol metabolite level did not show significant differences in the B/B ratio. The reason of such an outcome may be that this phylum-level indicator may not provide sufficient detail to capture meaningful differences in microbial communities at lower taxonomic levels. Thus, we propose the calculation of microbial indicators at a lower taxonomic level, specifically as ratios between selected bacterial families from the phyla Bacillota and Bacteroidota. The ratios of *Christensenellaceae* to *Rikenellaceae*, *Bacteroidaceae*, and *Prevotellaceae* were found to be significantly higher in the gut microbiota of roe deer with elevated cortisol levels, suggesting their potential role as specific microbial signatures associated with stress-related factors. The potential of *Christensenellaceae* as a marker of overall gut health and metabolic status that changes in response to various stressors has been earlier recognized^67^. Moreover, studies comparing the microbiota of ungulate populations inhabiting more favorable versus challenging environments (at low and high altitudes) found that *Christensenellaceae* were significantly enriched in the gut microbiomes of high-altitude ungulates^68^. On the other hand, this bacterial family has emerged as a potentially more consistent marker of microbiota resilience and host metabolic state via a methane-associated energy loss mechanism^69,70^. Accordingly, the question of whether a higher ratio of *Christensenellaceae* to *Rikenellaceae*, *Bacteroidaceae*, and *Prevotellaceae* in the gut microbiota of roe deer may reflect enhanced metabolic regulation and a greater ability to extract energy from fiber-rich forage (a critical adaptation in herbivores) remains open. If that were the case, this additional energy supply could be important for supporting the organism’s mobilization during stress conditions and mitigating loss of body mass^35^. Nonetheless, the utility of these indicators must be confirmed in further studies on roe deer or other cervid species.

The PERMANOVA results confirm that variation in microbiota composition among individuals is non-random. Among the environmental variables tested, area of origin emerged as the strongest predictor of microbiome structure, with season also contributing significantly, albeit to a lesser extent. Stress level was a significant factor as well, although its effect size was smaller than that of area and comparable to that of season. In additive models (e.g. Stress + Area) stress remained significant, but interaction terms involving stress were not significant, indicating that, within this dataset, stress does not interact synergistically with the other measured variables. A complementary analysis with stress treated as a continuous predictor confirmed that its influence on gut microbiota remains detectable. The stronger signal observed in the binary model may suggest a non-linear or threshold-dependent relationship, in which microbial composition shifts markedly only beyond certain stress levels. However, given the current lack of validated biological thresholds in wild cervids, this hypothesis remains speculative and warrants further investigation.

The gut microbiota functions as a complex and dynamic community, governed by numerous feedback-based interactions. As a result, it is generally more feasible to detect shifts at the community level than to identify clear, directional changes in the abundance of individual taxa—particularly when employing conservative, multi-stage selection methods such as those implemented in ANCOM-BC2. In our study, only a limited number of taxa were found to be statistically associated with the environmental and physiological variables analyzed. While variation between study sites and seasons appears predictable (largely due to diet composition being a primary driver of gut microbial structure^3,6,7^, the identification of bacterial groups associated with stress hormone levels remains less straightforward. In our analysis, only four bacterial groups exhibited a significant reduction in animals with elevated stress levels: *Barnesiellaceae*, *Succinivibrionaceae*, *Sutterellaceae*, and an uncultured rumen bacterium (4C0d_17).

The observed stress-associated decline in *Barnesiellaceae* aligns with previous findings indicating its sensitivity to physiological stress, with alterations in abundance linked to stress-induced behavioral and immunological changes. Higher levels of *Barnesiellaceae* have been associated with improved overall health status^71^, and recent studies have implicated this family in modulating individual responses to environmental stressors, particularly with respect to inflammation and gut permeability^72^. These findings suggest that this family may play a protective role in maintaining gut barrier integrity in stress-sensitive individuals. A reduction in its abundance could therefore reflect stress-induced dysfunction in intestinal permeability and the activation of mucosal immune responses. Similarly, reduced levels of *Succinivibrionaceae*, a family known for propionate production, have previously been reported in association with feed restriction and abrupt dietary changes. These microbial shifts are often accompanied by altered fermentation profiles and immune modulation^73,74^. Thus, the decreased abundance of *Succinivibrionaceae* observed in our study may be interpreted as a microbial signature of metabolic stress in the host, potentially related to disruptions in fermentation pathways in wild ungulates experiencing environmental pressure. Together, these two taxa (*Barnesiellaceae* and *Succinivibrionaceae*) may represent promising microbial biomarkers of physiological stress, particularly under conditions of compromised intestinal immune homeostasis.

Stress may also impact the abundance of *Sutterellaceae*, with potential implications for gut health and stress-related host behaviors; however, existing evidence remains sparse. *Sutterella* spp. have been reported to possess pro-inflammatory characteristics and the capacity to adhere to intestinal epithelial cells, indicating a possible immunomodulatory role^75^. In murine models exhibiting psychological stress and symptoms of mental disorders, increased abundance of *Parasutella* (a related genus) has been observed, with negative correlations to several fecal metabolites - pointing to disrupted metabolic pathways relevant to neurobehavioral regulation^76^. In contrast to these findings, our study revealed a significant reduction in *Sutterellaceae* abundance under conditions of elevated physiological stress. However, it is important to consider that our work focused on chronic environmental stress in free-ranging wild ungulates, which differs fundamentally from acute stress paradigms typically used in laboratory rodent studies. Moreover, *Sutterellaceae* abundance has been shown to vary strongly with host genetics rather than environmental factors such as diet, suggesting that its response to stress may differ across host species and ecological contexts^77^. These discrepancies emphasize the need for additional comparative studies to clarify the role of *Sutterellaceae* in stress-related microbiota dynamics. Finally, although no functional or ecological data are currently available for the uncultured rumen taxon 4C0d_17, its classification as a rumen-associated bacterium suggests that its decline may reflect impaired fiber fermentation capacity under metabolically stressful conditions^78,79^.

## Conclusions

This study provides new evidence that physiological stress, as reflected by elevated fecal cortisol metabolite concentrations, is associated with measurable shifts in the gut microbiota composition of free-ranging European roe deer inhabiting agricultural landscapes. Although alpha diversity metrics remained unaffected, beta diversity analyses revealed significant differences in microbial community structure between individuals with higher and lower stress levels. These differences persisted even after accounting for confounding variables such as geographic area and season, and became more pronounced in interaction models, suggesting a context-dependent modulation of microbiota by stress.

While the majority of bacterial taxa appeared resilient to stress-associated variation, a limited number of families showed consistent, significant changes in relative abundance. Notably, *Barnesiellaceae* and *Succinivibrionaceae* (both implicated in host immune regulation and fermentation processes) were depleted under higher stress conditions. The elevated *Christensenellaceae* to *Rikenellaceae*,* Bacteroidaceae* and *Prevotellaceae* ratios in stressed individuals points to its potential as a microbial biomarker of physiological stress. By identifying taxa that respond consistently to stress, this study advances the search for microbial indicators that could serve as non-invasive proxies for physiological state and environmental impact in wildlife. Such biomarkers may be particularly valuable in conservation biology, land-use planning, and monitoring the ecological effects of anthropogenic pressures on wild herbivore populations. Future studies should aim to validate these microbial signatures across broader spatial and temporal scales and explore their functional consequences for host health, reproduction, and fitness.

## Supplementary Information

Below is the link to the electronic supplementary material.

Supplementary Material 1

## Data Availability

All data used in this work were made available in the article (Supplementary Information file) and raw sequencing data were deposited in NCBI under accession number PRJNA1187881 (https://www.ncbi.nlm.nih.gov/bioproject/PRJNA1187881/).
