# Supplementary material for: Bacterial indicators of environmental stress in the gut microbiome of free-ranging European roe deer inhabiting agricultural landscapes
Source: Sci Rep. 2025 Aug 7;15:28876. doi: 10.1038/s41598-025-14933-w (PMC12332138; doi:10.1038/s41598-025-14933-w)
Supplement: Supplementary file 1 — Supplementary Material 1 [file 41598_2025_14933_MOESM1_ESM.docx]

**Table S1.** Characteristics of the roe deer included in the study, encompassing their place of origin, sex, age, fecal cortisol metabolite concentrations, and the classification into groups with lower and higher stress levels.

| **Sample ID** | **The study areas** | **Hunting district identification number** | **Sex** | **Age**  **[years]** | **Carcass weight**  **[kg]** | **Season** | **11-oxoetiocholanolone concentration**  **[ng/ml]** | **Assignment to a lower-or higher-stress group** |
| --- | --- | --- | --- | --- | --- | --- | --- | --- |
| IL11 | IŁŻA | 563 | M | 4 | 20 | Winter | 127.8 | Higher |
| IL18 | IŁŻA | 563 | F | 1.5 | 17 | Summer | 115.2 | Higher |
| IL21 | IŁŻA | 563 | F |  | 20 | Winter | 146.6 | Higher |
| IL28 | IŁŻA | 556 | F | 0.67 | 12 | Winter | 371.4 | Higher |
| IL31 | IŁŻA | 556 | F | 0.58 | 11 | Winter | 128.5 | Higher |
| IL32 | IŁŻA | 556 | F | 5 | 21 | Winter | 149.0 | Higher |
| IL33 | IŁŻA | 563 | F |  | 17 | Winter | 160.7 | Higher |
| IL38 | IŁŻA | 556 | F | 4 | 19 | Winter | 204.2 | Higher |
| IL40 | IŁŻA | 559 | F | 5 | 21 | Winter | 182.6 | Higher |
| IL42 | IŁŻA | 559 | F | 2 | 18 | Winter | 199.9 | Higher |
| RA8 | RAWA | 96 | F | 3 | 18 | Winter | 149.1 | Higher |
| RA10 | RAWA | 96 | M | 3 | 17 | Winter | 129.3 | Higher |
| RA11 | RAWA | 96 | M | 3.5 | 17 | Winter | 310.5 | Higher |
| RA14 | RAWA | 96 | M | 6 | 21 | Winter | 282.0 | Higher |
| RA15 | RAWA | 99 | M | 4 | 18 | Winter | 164.2 | Higher |
| RA16 | RAWA | 99 | F | 3.5 | 17 | Winter | 278.9 | Higher |
| RA19 | RAWA | 96 | F | 4 | 17 | Winter | 161.6 | Higher |
| WE3 | WĘGRÓW | 336 | M | 4 | 17 | Winter | 280.6 | Higher |
| WE4 | WĘGRÓW | 283 | M | 5 | 15 | Winter | 212.2 | Higher |
| WE7 | WĘGRÓW | 283 | F | 1 | 12 | Winter | 303.5 | Higher |
| WE11 | WĘGRÓW | 316 | M |  | 17 | Winter | 149.7 | Higher |
| WE12 | WĘGRÓW | 316 | F | 4 | 21 | Winter | 314.8 | Higher |
| WE15 | WĘGRÓW | 283 | F | 2 | 17 | Winter | 140.7 | Higher |
| WE17 | WĘGRÓW | 283 | M | 1 | 12 | Winter | 322.1 | Higher |
| WE26 | WĘGRÓW | 316 | M | 8 | 18 | Winter | 176.2 | Higher |
| WE27 | WĘGRÓW | 336 | M | 4 | 17 | Winter | 189.3 | Higher |
| WE28 | WĘGRÓW | 283 | F | 4 | 26 | Winter | 105.6 | Lower |
| WE30 | WĘGRÓW | 283 | F | 3 | 24 | Winter | 106.5 | Lower |
| IL10 | IŁŻA | 559 | F | 4 | 20 | Winter | 94.3 | Lower |
| IL12 | IŁŻA | 562 | F | 2 | 19 | Summer | 54.4 | Lower |
| IL14 | IŁŻA | 563 | F | 4 | 20 | Winter | 21.5 | Lower |
| IL20 | IŁŻA | 563 | F | 4 | 18 | Winter | 57.7 | Lower |
| IL26 | IŁŻA | 559 | F | 3 | 17 | Winter | 49.9 | Lower |
| IL27 | IŁŻA | 559 | F | 2 | 16 | Summer | 70.2 | Lower |
| IL34 | IŁŻA | 563 | F | 4 | 17 | Winter | 58.8 | Lower |
| IL37 | IŁŻA | 559 | F | 3 | 21 | Winter | 49.9 | Lower |
| IL39 | IŁŻA | 556 | F |  | 20 | Winter | 57.7 | Lower |
| RA21 | RAWA | 96 | F | 4 | 17 | Winter | 33.7 | Lower |
| RA22 | RAWA | 96 | F |  | 17 | Summer | 85.2 | Lower |
| RA35 | RAWA | 145 | F | 3 | 16 | Winter | 68.0 | Lower |
| RA36 | RAWA | 137 | F | 4 | 18.7 | Winter | 22.9 | Lower |
| RA37 | RAWA | 137 | F | 4 | 15.6 | Summer | 19.2 | Lower |
| RA39 | RAWA | 99 | M | 5 | 18 | Summer | 94.4 | Lower |
| WE9 | WĘGRÓW | 316 | F | 2 | 18 | Summer | 31.8 | Lower |
| WE10 | WĘGRÓW | 316 | F | 1 | 12 | Summer | 86.7 | Lower |
| WE13 | WĘGRÓW | 316 | M | 1 | 12 | Winter | 84.3 | Lower |
| WE14 | WĘGRÓW | 283 | F | 2 | 18 | Winter | 53.2 | Lower |
| WE16 | WĘGRÓW | 283 | F | 3 | 19 | Winter | 20.3 | Lower |
| WE18 | WĘGRÓW | 283 | F | 4 | 20 | Winter | 40.6 | Lower |
| WE19 | WĘGRÓW | 283 | F | 1 | 12 | Winter | 97.6 | Lower |
| WE29 | WĘGRÓW | 283 | F | 5 | 24 | Winter | 17.9 | Lower |
| WE31 | WĘGRÓW | 283 | F | 2 | 19 | Winter | 79.9 | Lower |
| WE32 | WĘGRÓW | 283 | F | 2 | 17 | Summer | 20.1 | Lower |
| WE33 | WĘGRÓW | 316 | F | 4 | 20 | Winter | 38.9 | Lower |

**Table S2.** Summary on quality control of reads, data preprocessing and ASV selecting

| **Sample ID** | **Raw Reads** | **Clean Reads** | **Non-chimeric Reads** | | **Feature sequences** | **ASVs** |
| --- | --- | --- | --- | --- | --- | --- |
| IL10 | 159622 | 149910 | 136615 | 136436 | | 869 |
| IL11 | 160203 | 149697 | 130184 | 129950 | | 1175 |
| IL12 | 160080 | 150761 | 122781 | 122134 | | 1342 |
| IL14 | 160223 | 151255 | 104340 | 103956 | | 1150 |
| IL18 | 159926 | 150405 | 133153 | 132854 | | 1398 |
| IL20 | 159893 | 149732 | 106720 | 106161 | | 1713 |
| IL21 | 159844 | 150013 | 105016 | 104328 | | 1927 |
| IL26 | 159981 | 150041 | 129471 | 129171 | | 784 |
| IL27 | 159921 | 150068 | 94511 | 93728 | | 2192 |
| IL28 | 160075 | 150790 | 134145 | 133940 | | 1048 |
| IL31 | 160056 | 150021 | 120162 | 119766 | | 1335 |
| IL32 | 160160 | 150396 | 120839 | 120108 | | 2811 |
| IL33 | 160056 | 150195 | 123219 | 122705 | | 1300 |
| IL34 | 160001 | 150070 | 93135 | 92356 | | 1804 |
| IL37 | 159843 | 150420 | 127962 | 127574 | | 1094 |
| IL38 | 160034 | 150130 | 139885 | 139641 | | 1037 |
| IL39 | 159979 | 150440 | 93082 | 92478 | | 1646 |
| IL40 | 160203 | 150713 | 114367 | 113766 | | 1955 |
| IL42 | 159768 | 150026 | 116167 | 115602 | | 1346 |
| RA10 | 160147 | 150915 | 104538 | 103996 | | 1712 |
| RA11 | 159790 | 149445 | 123441 | 122808 | | 1403 |
| RA14 | 159723 | 150187 | 128756 | 128301 | | 1331 |
| RA15 | 160148 | 150164 | 99020 | 98392 | | 1841 |
| RA16 | 159986 | 149783 | 131514 | 131169 | | 897 |
| RA19 | 159584 | 149681 | 123095 | 122660 | | 1133 |
| RA21 | 160008 | 150433 | 96248 | 95578 | | 1076 |
| RA22 | 159990 | 149888 | 106834 | 106377 | | 1196 |
| RA35 | 159972 | 150237 | 99750 | 98928 | | 2223 |
| RA36 | 159478 | 149270 | 126014 | 125578 | | 1216 |
| RA37 | 159810 | 150301 | 119102 | 118675 | | 1111 |
| RA39 | 160158 | 150586 | 121747 | 121191 | | 1272 |
| RA8 | 159982 | 149511 | 97147 | 96389 | | 1998 |
| WE10 | 159945 | 149580 | 128566 | 128261 | | 855 |
| WE11 | 160010 | 150423 | 109506 | 109111 | | 1263 |
| WE12 | 160283 | 149998 | 101758 | 101397 | | 1128 |
| WE13 | 160160 | 151193 | 123348 | 122959 | | 1556 |
| WE14 | 159609 | 149299 | 119980 | 119465 | | 989 |
| WE15 | 160077 | 150301 | 119125 | 118786 | | 781 |
| WE16 | 160179 | 150403 | 126507 | 126214 | | 778 |
| WE17 | 160268 | 150494 | 127261 | 126882 | | 792 |
| WE18 | 159587 | 149429 | 98102 | 97599 | | 1347 |
| WE19 | 160066 | 150709 | 115110 | 114760 | | 1060 |
| WE26 | 159945 | 149394 | 141901 | 141761 | | 677 |
| WE27 | 160420 | 150402 | 103822 | 103207 | | 1844 |
| WE28 | 159804 | 150231 | 130497 | 130229 | | 842 |
| WE29 | 159948 | 149844 | 135219 | 134992 | | 875 |
| WE3 | 159885 | 150182 | 123371 | 123016 | | 1070 |
| WE30 | 160147 | 149627 | 115559 | 114956 | | 1129 |
| WE31 | 159996 | 150341 | 142477 | 142331 | | 559 |
| WE32 | 160206 | 149915 | 111246 | 110618 | | 1914 |
| WE33 | 159954 | 150849 | 135401 | 135097 | | 1043 |
| WE4 | 160191 | 150750 | 121882 | 121462 | | 1198 |
| WE7 | 159990 | 150096 | 118329 | 118014 | | 1335 |
| WE9 | 160189 | 150831 | 99234 | 98628 | | 1870 |
|  |  |  |  |  | |  |


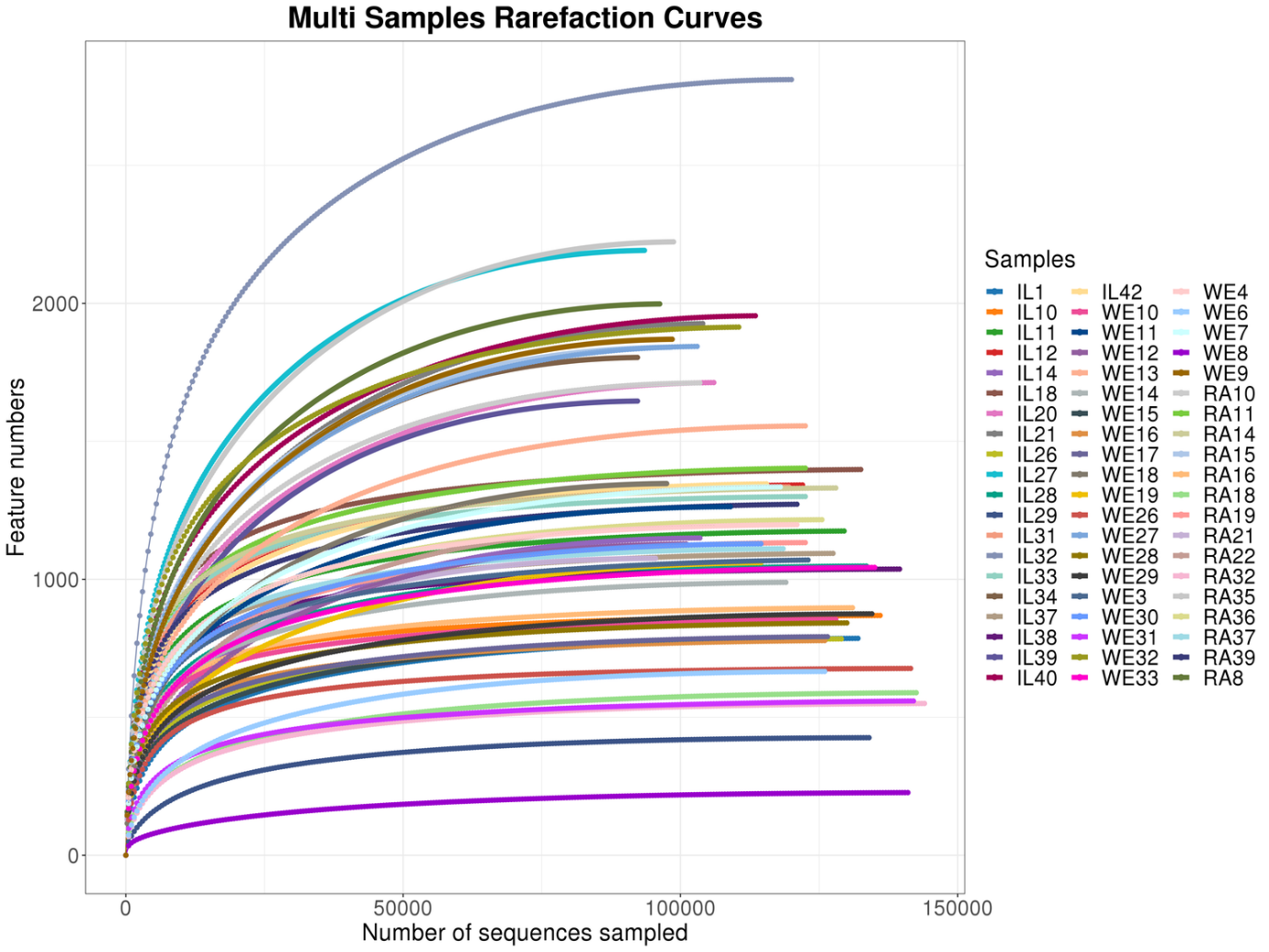


**Figure S1**. Rarefaction curves


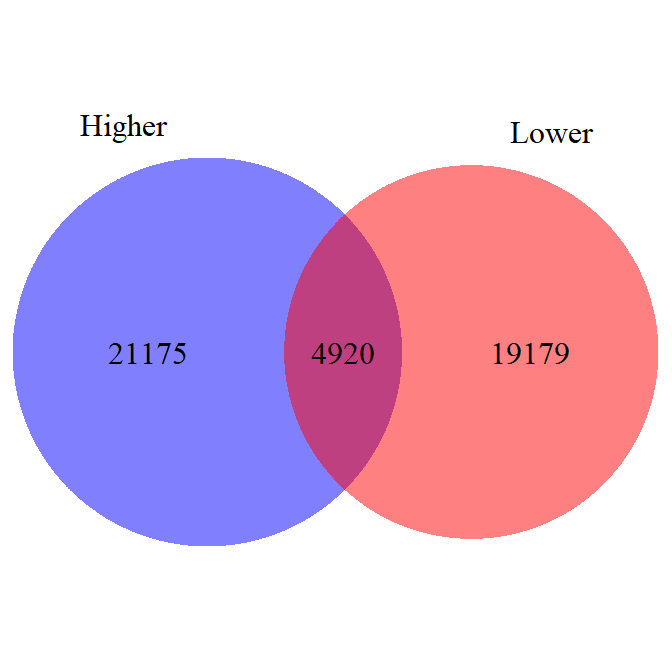


**Figure S2.** Diagram showing overlapping and specific ASVs among groups of bacterial microbiota of roe deer differing in fecal cortisol level. Abbreviations: Lower – the group with low cortisol metabolite concentration, Higher – the group with elevated cortisol metabolite concentration, N= 28 and N=26, respectively.

**Table S3.** Summary on taxonomic annotation of feature sequences

| **Sample** |  | **Phylum** | **Class** | **Order** | **Family** | **Genus** |
| --- | --- | --- | --- | --- | --- | --- |
| IL10 |  | 17 | 24 | 43 | 74 | 162 |
| IL11 |  | 15 | 21 | 45 | 87 | 176 |
| IL12 |  | 15 | 25 | 48 | 88 | 210 |
| IL14 |  | 13 | 19 | 45 | 73 | 168 |
| IL18 |  | 18 | 28 | 53 | 97 | 192 |
| IL20 |  | 16 | 25 | 58 | 99 | 208 |
| IL21 |  | 16 | 25 | 59 | 104 | 199 |
| IL26 |  | 12 | 17 | 35 | 71 | 163 |
| IL27 |  | 21 | 35 | 74 | 128 | 245 |
| IL28 |  | 12 | 17 | 43 | 76 | 179 |
| IL31 |  | 13 | 18 | 45 | 84 | 176 |
| IL32 |  | 33 | 73 | 172 | 310 | 587 |
| IL33 |  | 16 | 26 | 58 | 110 | 210 |
| IL34 |  | 17 | 25 | 58 | 94 | 192 |
| IL37 |  | 15 | 21 | 49 | 86 | 188 |
| IL38 |  | 18 | 30 | 64 | 107 | 198 |
| IL39 |  | 16 | 24 | 59 | 104 | 198 |
| IL40 |  | 14 | 25 | 51 | 90 | 194 |
| IL42 |  | 16 | 25 | 52 | 94 | 198 |
| RA10 |  | 15 | 26 | 50 | 89 | 180 |
| RA11 |  | 13 | 19 | 47 | 81 | 193 |
| RA14 |  | 13 | 18 | 45 | 83 | 181 |
| RA15 |  | 14 | 25 | 55 | 93 | 190 |
| RA16 |  | 13 | 19 | 39 | 67 | 158 |
| RA19 |  | 13 | 19 | 41 | 79 | 181 |
| RA21 |  | 12 | 19 | 36 | 60 | 148 |
| RA22 |  | 14 | 21 | 47 | 82 | 170 |
| RA35 |  | 21 | 34 | 67 | 118 | 222 |
| RA36 |  | 15 | 24 | 52 | 93 | 176 |
| RA37 |  | 13 | 22 | 45 | 77 | 187 |
| RA39 |  | 14 | 21 | 44 | 80 | 174 |
| RA8 |  | 15 | 23 | 54 | 92 | 195 |
| WE10 |  | 13 | 19 | 39 | 64 | 154 |
| WE11 |  | 15 | 21 | 46 | 79 | 181 |
| WE12 |  | 10 | 15 | 35 | 63 | 144 |
| WE13 |  | 16 | 27 | 57 | 105 | 213 |
| WE14 |  | 14 | 21 | 42 | 75 | 165 |
| WE15 |  | 12 | 19 | 37 | 64 | 150 |
| WE16 |  | 16 | 21 | 42 | 71 | 148 |
| WE17 |  | 12 | 16 | 32 | 64 | 149 |
| WE18 |  | 15 | 22 | 50 | 79 | 183 |
| WE19 |  | 15 | 20 | 48 | 83 | 179 |
| WE26 |  | 14 | 21 | 39 | 66 | 149 |
| WE27 |  | 18 | 31 | 62 | 107 | 207 |
| WE28 |  | 11 | 17 | 41 | 70 | 157 |
| WE29 |  | 16 | 23 | 53 | 97 | 192 |
| WE3 |  | 13 | 21 | 47 | 79 | 167 |
| WE30 |  | 13 | 18 | 45 | 82 | 172 |
| WE31 |  | 13 | 20 | 42 | 71 | 155 |
| WE32 |  | 15 | 23 | 50 | 95 | 222 |
| WE33 |  | 15 | 22 | 51 | 93 | 222 |
| WE4 |  | 13 | 20 | 47 | 88 | 181 |
| WE7 |  | 13 | 20 | 46 | 80 | 178 |
| WE9 |  | 11 | 18 | 41 | 73 | 176 |

**A.**


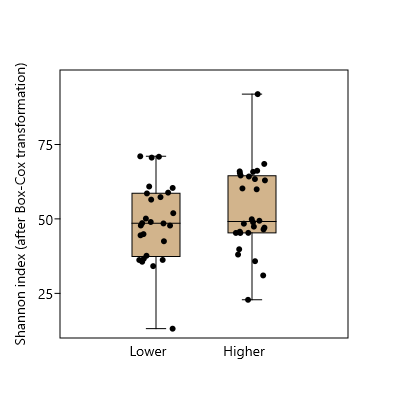


**B.**


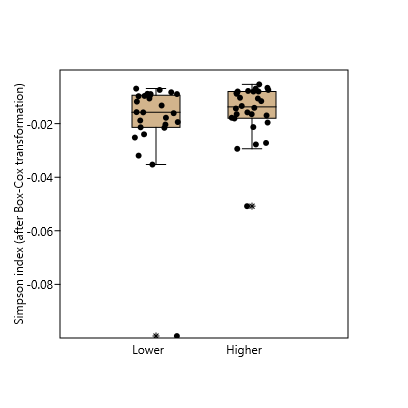


**Figure S3.** Shannon (A) and Simpson (B) indices groups of bacterial microbiota of roe deer differing in fecal cortisol level. Abbreviations: Lower – the group with low cortisol metabolite concentration, Higher – the group with elevated cortisol metabolite concentration, N= 28 and N=26, respectively. t=1.49, p=0.15 t=1.12, p=0.3

Table S4. Results of PERMANOVA assessing the effects of four selected factors (Area, log-transformed cortisol metabolite concentration - logConc, Season, and Weight) and their interactions on beta diversity (Bray–Curtis distance) of the gut microbiota in European roe deer. The table includes all models in which at least one term had a statistically significant effect (p < 0.05). For each Model (full formula fitted in PERMANOVA), the following parameters are reported: Total R² (variance explained by the entire model), Total F (F-statistic for the whole model), Model p (permutation p-value for the whole model), Term (individual factor or interaction being tested), Df (degrees of freedom for the term), SS partial (marginal sum of squares for the term), R² partial (unique proportion of variance explained by the term), F partial (F-statistic for the term after accounting for others), and Term p (permutation p-value for the term). In interaction models, only the highest-order interaction term is presented.

Cortisol metabolite concentration was log-transformed using log10(x + 1) prior to analysis.

| **Model** | **Total** | **Total** | **Model** | **Term** | **Df** | **SS** | **R2** | **F** | **Term** |
| --- | --- | --- | --- | --- | --- | --- | --- | --- | --- |
|  | **R²** | **F** | **p** |  |  | **(partial)** | **(partial)** | **(partial)** | **p** |
| logConc | 0.034 | 1.827 | 0.070 | logConc |  |  |  |  |  |
| Area | 0.120 | 3.473 | <0.001 | Area |  |  |  |  |  |
| Season | 0.043 | 2.342 | 0.024 | Season |  |  |  |  |  |
| Weight | 0.013 | 0.660 | 0.728 | Weight |  |  |  |  |  |
| logConc + Area | 0.151 | 2.957 | <0.001 | logConc | 1 | 0.137 | 0.031 | 1.812 | 0.071 |
|  |  |  |  | Area | 2 | 0.521 | 0.117 | 3.437 | <0.001 |
| logConc + Season | 0.066 | 1.794 | 0.040 | logConc | 1 | 0.101 | 0.023 | 1.234 | 0.240 |
|  |  |  |  | Season | 1 | 0.142 | 0.032 | 1.736 | 0.084 |
| logConc + Weight | 0.048 | 1.278 | 0.196 | logConc | 1 | 0.157 | 0.035 | 1.884 | 0.068 |
|  |  |  |  | Weight | 1 | 0.062 | 0.014 | 0.739 | 0.647 |
| Area + Season | 0.158 | 3.131 | <0.001 | Area | 2 | 0.514 | 0.115 | 3.417 | <0.001 |
|  |  |  |  | Season | 1 | 0.171 | 0.038 | 2.274 | 0.033 |
| Area + Weight | 0.131 | 2.517 | <0.001 | Area | 2 | 0.530 | 0.119 | 3.415 | <0.001 |
|  |  |  |  | Weight | 1 | 0.051 | 0.011 | 0.653 | 0.743 |
| Season + Weight | 0.056 | 1.502 | 0.096 | Season | 1 | 0.192 | 0.043 | 2.327 | 0.025 |
|  |  |  |  | Weight | 1 | 0.056 | 0.013 | 0.677 | 0.722 |
| logConc + Area + Season | 0.180 | 2.691 | <0.001 | logConc | 1 | 0.098 | 0.022 | 1.310 | 0.203 |
|  |  |  |  | Area | 2 | 0.511 | 0.114 | 3.418 | <0.001 |
|  |  |  |  | Season | 1 | 0.131 | 0.029 | 1.758 | 0.080 |
| logConc + Area + Weight | 0.162 | 2.375 | 0.001 | logConc | 1 | 0.139 | 0.031 | 1.823 | 0.076 |
|  |  |  |  | Area | 2 | 0.512 | 0.115 | 3.354 | 0.001 |
|  |  |  |  | Weight | 1 | 0.052 | 0.012 | 0.686 | 0.711 |
| logConc + Season + Weight | 0.079 | 1.425 | 0.097 | logConc | 1 | 0.103 | 0.023 | 1.255 | 0.240 |
|  |  |  |  | Season | 1 | 0.139 | 0.031 | 1.684 | 0.099 |
|  |  |  |  | Weight | 1 | 0.058 | 0.013 | 0.708 | 0.694 |
| Area + Season + Weight | 0.170 | 2.501 | <0.001 | Area | 2 | 0.509 | 0.114 | 3.360 | 0.000 |
|  |  |  |  | Season | 1 | 0.171 | 0.038 | 2.260 | 0.033 |
|  |  |  |  | Weight | 1 | 0.051 | 0.011 | 0.671 | 0.733 |
| logConc * Area | 0.198 | 2.363 | <0.001 | logConc:Area | 2 | 0.209 | 0.047 | 1.401 | 0.130 |
| logConc * Season | 0.077 | 1.398 | 0.107 | logConc:Season | 1 | 0.052 | 0.012 | 0.633 | 0.756 |
| logConc * Weight | 0.078 | 1.415 | 0.100 | logConc:Weight | 1 | 0.136 | 0.031 | 1.658 | 0.101 |
| Area * Season | 0.195 | 2.326 | <0.001 | Area:Season | 2 | 0.165 | 0.037 | 1.099 | 0.335 |
| Area * Weight | 0.165 | 1.901 | 0.002 | Area:Weight | 2 | 0.152 | 0.034 | 0.980 | 0.454 |
| Season * Weight | 0.074 | 1.331 | 0.133 | Season:Weight | 1 | 0.082 | 0.018 | 0.990 | 0.411 |
| logConc * Area * Season | 0.284 | 1.706 | 0.001 | logConc:Area:Season | 1 | 0.070 | 0.016 | 0.944 | 0.457 |
| logConc * Area * Weight | 0.287 | 1.534 | 0.004 | logConc:Area:Weight | 2 | 0.116 | 0.026 | 0.762 | 0.716 |
| logConc * Season * Weight | 0.156 | 1.210 | 0.154 | logConc:Season:Weight | 1 | 0.075 | 0.017 | 0.915 | 0.481 |
| Area * Season * Weight | 0.281 | 1.682 | 0.002 | Area:Season:Weight | 1 | 0.055 | 0.012 | 0.735 | 0.658 |


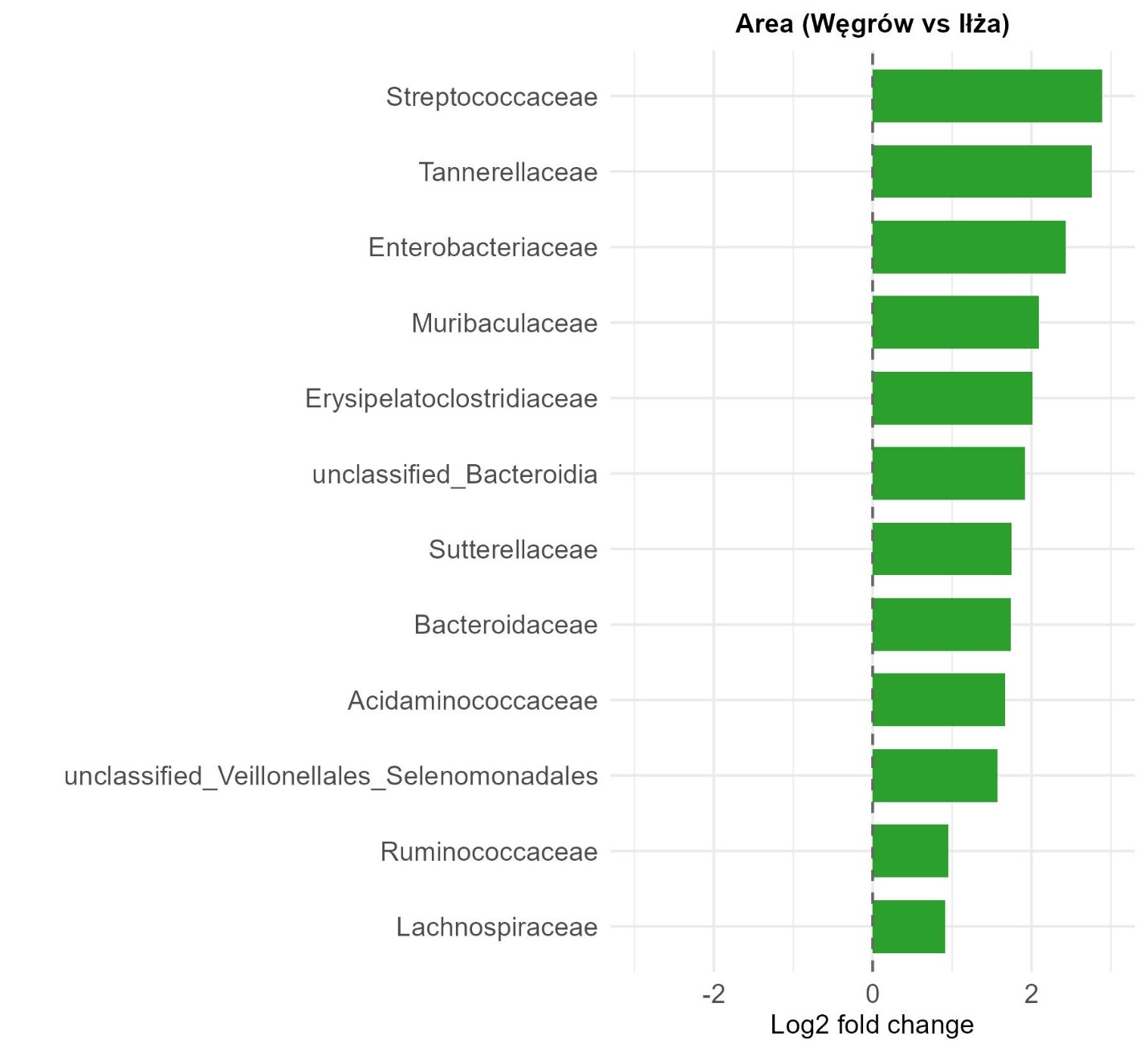


**Figure S4**. Differences in bacterial family-level composition between roe deer from Węgrów and Iłża. Bar plot displays families with significantly higher relative abundances in individuals sampled from Węgrów compared to Iłża, based on ANCOM-BC2 results (FDR-adjusted q < 0.05; passed signal sensitivity threshold). All shown taxa were more abundant in Węgrów. Bars represent log2 fold changes (Węgrów vs Iłża).


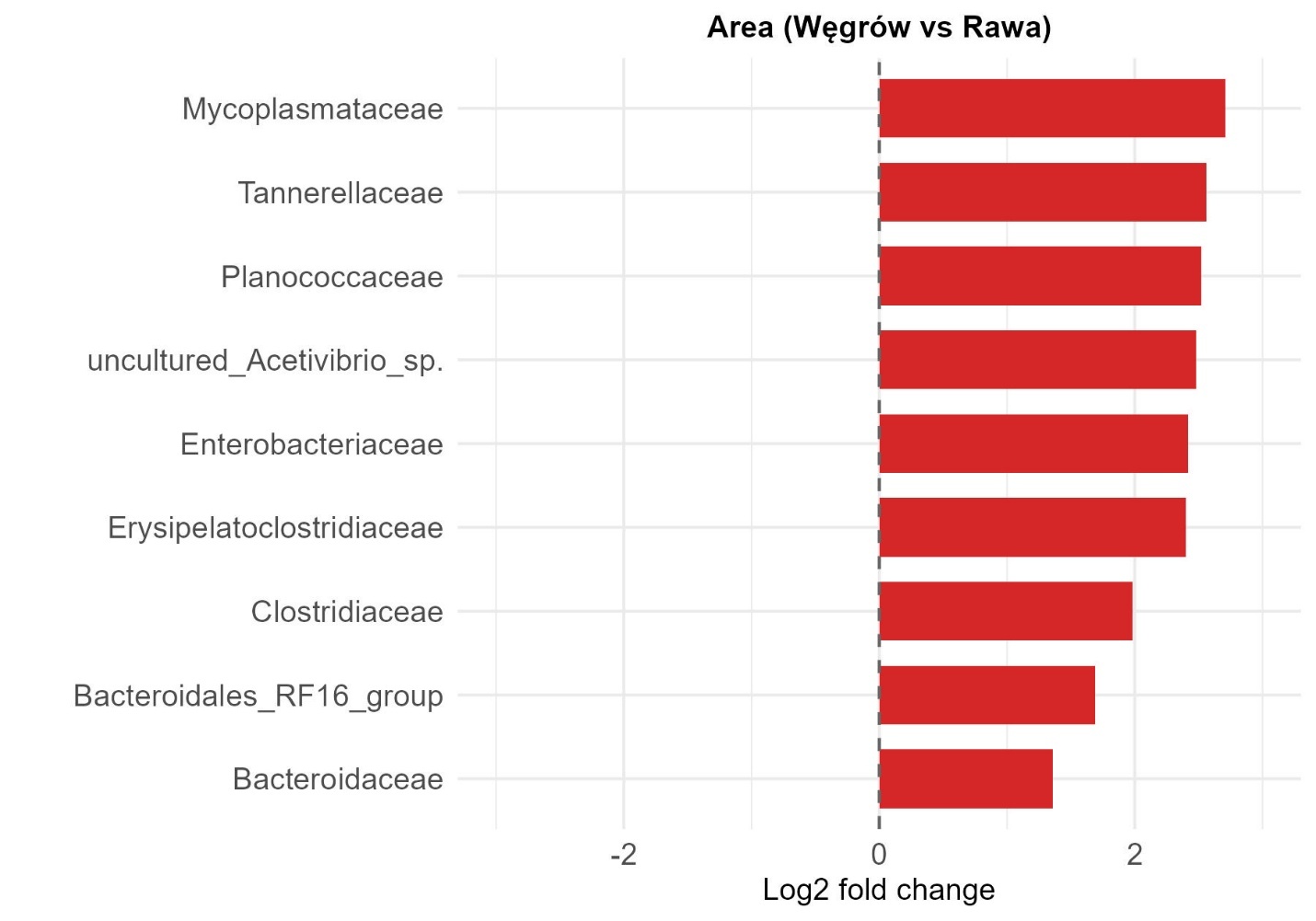


**Figure S5**. Differentially abundant bacterial families between roe deer from Węgrów and Rawa. Bar plot shows families with significantly increased relative abundance in samples from Węgrów compared to Rawa, as identified by ANCOM-BC2 (FDR-adjusted q < 0.05; passed signal sensitivity threshold). All displayed taxa were more abundant in individuals from Węgrów. Bars indicate log2 fold changes (Węgrów vs Rawa).
